# Application of IRI Visualization to Terahertz Vibrational Spectroscopy of Hydroxybenzoic Acid Isomers

**DOI:** 10.3390/ijms241310417

**Published:** 2023-06-21

**Authors:** Shan Tu, Wentao Zhang, Yuan Tang, Yuanpeng Li, Junhui Hu

**Affiliations:** 1 School of Electronic Engineering and Automation, Guilin University of Electronic Technology, Guilin 541004, China; ts@mailbox.gxnu.edu.cn (S.T.); tangyuan@guet.edu.cn (Y.T.); 2 Guangxi Key Laboratory of Nuclear Physics and Technology, Guangxi Normal University, Guilin 541004, China; yuanpengli@gxnu.edu.cn; 3 Guangxi Key Laboratory of Optoelectronic Information Processing, Guilin University of Electronic Technology, Guilin 541004, China

**Keywords:** terahertz spectrum, isomer, density functional theory, vibrational mode, interaction region indicator

## Abstract

The characteristic absorption spectra of three positional isomers of hydroxybenzoic acid are measured using a terahertz time-domain spectroscopy system (THz-TDS) in the 0.6–2.0 THz region at room temperature. Significant differences in their terahertz spectra are discovered, which indicates that THz-TDS is an effective means to identify positional isomers. In order to simulate their spectra, the seven molecular clusters of 2-, 3-, and 4-hydroxybenzoic acid (2-, 3-, and 4-HA) are calculated using the DFT-D3 method. Additionally, the potential energy distribution (PED) method is used to analyze their vibration modes. The analysis indicates that the vibration modes of 2-HA are mainly out-of-plane angle bending and bond angle bend in plane. The vibration modes of 3-HA are mainly bond length stretch and dihedral angle torsion. The vibration modes of 4-HA are mainly bond angle bend in plane and dihedral angle torsion. Interaction region indicator (IRI) analysis is used to visualize the location and type of intermolecular interactions in 2-, 3-, and 4-HA crystals. The results show that the weak interaction type of 2-, 3-, and 4-HA is dominated by van der Waals (vdW) interaction. Therefore, we can confirm that terahertz spectroscopy detection technology can be used as an effective means to identify structural isomers and detect the intermolecular interactions in these crystals. In addition, it can explain the absorption mechanism of terahertz waves interacting with matter.

## 1. Introduction

When exploring the synthesis and decomposition of organic substances, the covalent bond between atoms is the focus of our attention, but there are not only covalent bonds in the organic molecular system, but also non-covalent bonds that are usually easily overlooked due to their weak interactions. These weak interactions, including intermolecular and intramolecular weak interactions, play an extremely important role in organic molecular systems and chemical synthesis. These weak interactions contain important physical and chemical information, which is of great significance for analyzing the internal structure of organic molecular systems and the subtle differences between molecules. 2-HA (a fat-soluble organic acid), which exists in natural willow bark, white bead leaves, and sweet birch trees, is an important fine chemical raw material and can be used for the preparation of aspirin and other drugs [1], chemical reaction fluorescent indicator Agent [2], complexing agent for electroplating or electroless plating [3], and a trace amount of preservative used in food [4]. 3-HA is a positional isomer of 2-HA, which can be used as a preservative for industrial products [5], ion exchangers for chemical synthesis [6], intermediates for the preparation of pharmaceuticals [7], and azo dyes [8]. 4-HA is also a positional isomer of 2-HA and mainly used as a preservative in medicines and cosmetics [9,10]. Many researchers have done a lot of research on 2-, 3-, and 4-HA. Boczar M [11] proposed a theoretical model of the infrared spectra of the 2-HA hydrogen bond stretching zone, and theoretically calculated the infrared spectra of the O-H stretching zone of 2-HA; the results showed that the experimental spectra were in good agreement with the theoretical spectra. However, in the detection of organic molecules via infrared spectroscopy, the quantitative analysis error is large, the sensitivity is low, and the spectrum analysis mainly depends on experience. On the other hand, the THz wave is an electromagnetic wave in the transition band between millimeter wave and infrared. The wave number range is 3.33–333 cm^−1^, and the frequency range is 0.1–10 THz [12,13]. Although the THz wave is the least known and developed wave so far, it has the unique advantages of low energy, strong penetrability, and good transient and high resolution in optical detection. Ding L [14] reported the two THz spectrum absorption characteristics of 2-HA and the three THz spectrum absorption characteristics of sodium salicylate. It has been shown that 2-HA and sodium salicylate can easily be distinguished using spectroscopy based on their unique THz, which may be due to the differences in their intramolecular and intermolecular structures. It indicates that the THz spectrum can be used to detect organic molecules.

In recent years, more and more researchers have simulated the spectral properties of positional isomers through density functional theory, which shows that the absorption characteristics of 2-HA are mainly derived from intermolecular interactions, and the absorption of sodium salicylate is mainly derived from intramolecular vibrations. Evangelisti L [15] used free jet millimeter-wave absorption spectroscopy to study the rotational spectra of 2-HA and three kinds of deuterides, and the results showed that the presence of OH groups in the adjacent position of benzoic acid did not affect the structure and stability of adjacent carboxyl groups. Vulpius D [16] used ultrashort laser pulse time-resolved laser-induced fluorescence spectroscopy to study the excited state proton transfer of 3-HA and 4-HA and discovered that new species only exist in excited states, which are caused by the temporary reversible annihilation of the aromatic bond system. Wang Q [17] tested the vibrational spectra of pyrazinamide (PZA), 3-HA, and their co-crystals using THz-TDS and Raman vibrational spectroscopy techniques, and the characteristic absorption peaks of the co-crystals were at 0.81, 1.47, and 1.61 THz, respectively. Additionally, through comparing the results of the simulated DFT frequency with the experimental vibration spectrum, the vibration mode of the eutectic is determined. Lepodise [18] studied benzoic acid and its derivatives 2-HA and 3-HA in the 6.06–15.15 THz spectral range, and the spectrum showed obvious absorption characteristics. The absorption curves of the experiment and the numerical simulation are basically in agreement. Choi Y [19] confirmed the coordination effect of 4-HA in polyethylene oxide via Fourier transform infrared spectroscopy. And further thermogravimetric analysis confirmed that 4-HA has strong stability in the polymer matrix. Brandan [20] measured the infrared and Raman spectra of 4-HA, then used the DFT (B3LYP) method to optimize the geometric structure of the monomer and dimer of 4-HA under the 6-31G*, D95**, and 6-311++G** base sets, respectively. The recorded vibration spectrum and theoretical calculation results show that 4-HA dimer has a stable conformation. Additionally, the formation of hydrogen bonds was studied from the perspective of charge density using the AIM program and NBO calculation. 

The research mentioned above confirms that THz spectroscopy technology is feasible for detection and has a relatively obvious absorption on 2-, 3-, and 4-HA [21]. In addition, THz waves have been proved to be universally feasible for the identification and classification of organics [22,23,24]. In the paper, the PED method was used to analyze the vibration modes of these three isomers, and the absorption peaks of the experimental spectra were identified. Moreover, a new method, which is a visual model based on IRI, is proposed to analyze the weak intramolecular and intermolecular interactions of 2-, 3-, and 4-HA. Through the visual analysis of IRI, the weakly interacting regions, composition, and strength of 2-, 3-, and 4-HA clusters can be intuitively captured. The results show that THz spectroscopy technology combined with stoichiometric simulation will help us further explore the internal structure and interaction characteristics of more organic isomers and metal organic framework structures (MOFs).

## 2. Results and Discussion

### 2.1. The Simulated THz Spectra of 2-, 3-, and 4-HA

**Figure 1:**
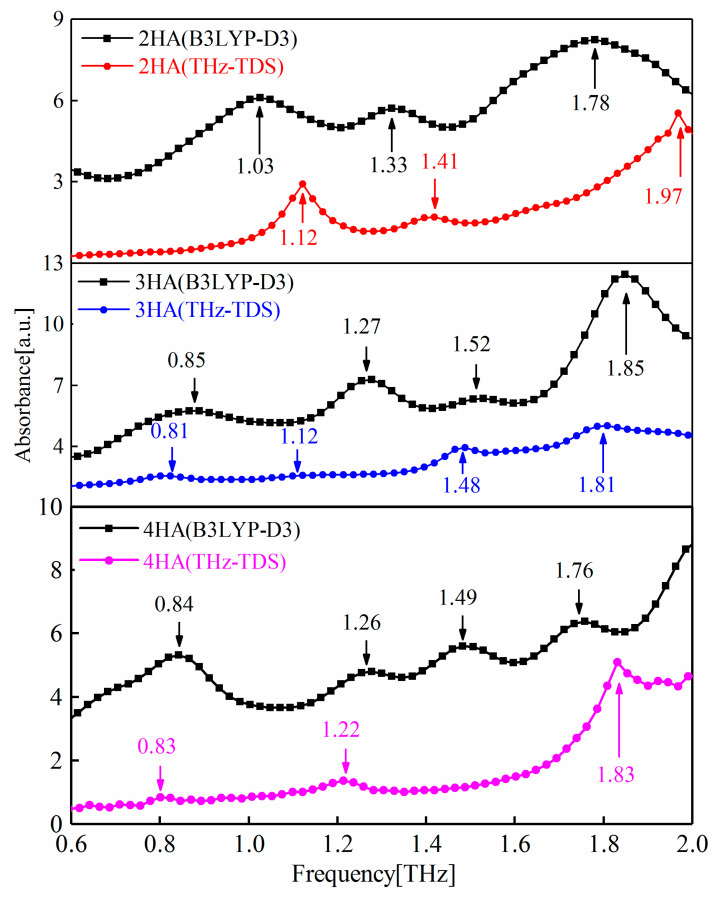
Comparison between the THz-TDS obtained and B3LYP-D3 simulated spectra of 2-, 3-, and 4-HA. The simulated spectrum has been shifted up vertically for clarity.

Assuming that the thermal effects on the samples could be ignored, the calculated spectra are shown in Figure 1. Obviously, differences in the experimentally obtained and calculated spectra are apparent in the spectra presented. The differences could be attributed to the dynamic simulation carried out with the 2-, 3-, and 4-HA molecules at the default temperature of 0 K, while the experiments were performed at the temperature of 295 K [25]. Furthermore, due to fluctuations in power, changes in the vibrational state of the environment, and changes in the air pressure, experimental results are often influenced by the system (or instrument) error and environmental noise.

Figure 1 shows the THz-TDS-obtained and B3LYP-D3-simulated spectra of 2-, 3-, and 4-HA. Evidently, the calculated peaks at 1.03 THz and 1.33 THz correspond to the measured peaks at 1.12 THz and 1.41 THz in 2-HA, while 0.85 THz, 1.52 THz, and 1.85 THz of calculated peaks correspond with the measured peaks at 0.81 THz, 1.48 THz, and 1.81 THz, respectively, in 3-HA. Similarly, in 4-HA, 0.84 THz, 1.26 THz, and 1.76 THz correspond with 0.83 THz, 1.22 THz, and 1.83 THz of the measured peaks, respectively.

### 2.2. Analysis of the Vibrational Modes

**Figure 2:**
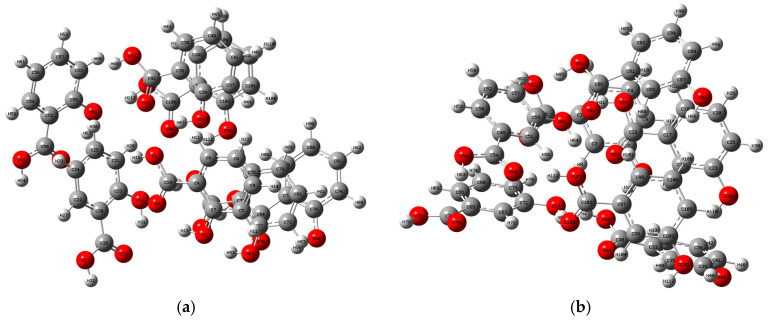

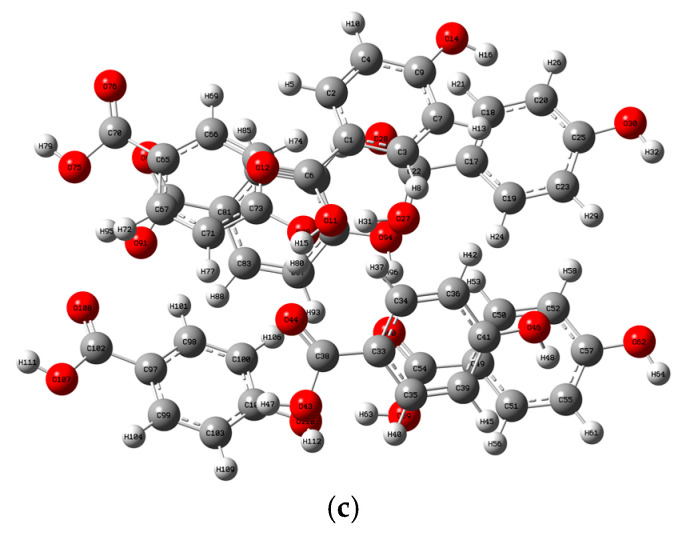
Index of atomic number corresponding to the optimized geometric structure for (**a**) 2-HA cluster of seven molecules, (**b**) 3-HA cluster of seven molecules, and (**c**) 4-HA cluster of seven molecules. Atomic elements in gray, white, and red represent carbon, hydrogen, and oxygen atoms, respectively.

In order to further study the relationship between THz absorption peaks and the vibration modes of 2-, 3-, and 4-HA molecules, simulation data analysis is performed via PED [26], and the results of the corresponding relationship between each absorption peak and the vibration mode are shown in Table 1. The atomic number indexes of the optimized geometries of 2-HA, 3-HA, and 4-HA are shown in Figure 2a–c, in that order. The experimental absorption peak of 2-HA at 1.12 THz corresponds to the simulated absorption peak at 1.03 THz, where the absorption peak is composed of O_(1)_-H_(16)_ and C_(25)_-C_(21)_-H_(16)._ The vibration modes caused by two H-bond interactions are all classified as out-of-plane angle bending. The experimental absorption peak at 1.41 THz corresponds to the simulated absorption peak at 1.33 THz, where the absorption peak is composed of H_(48)_-C_(53)_-C_(57)_ and C_(10)_-C_(103)_-O_(98)_-H_(87)_ caused by two H-bond interactions; the vibration modes are respectively attributed to bond angle bend in plane and dihedral angle torsion. The experimental absorption peak at 1.97 THz has no corresponding simulated peak, and the simulated absorption peak at 1.78 THz has no corresponding experimental peak. The absorption peaks at these two places are both classified as intermolecular interaction. The experimental absorption peak of 3-HA at 1.48 THz corresponds to the simulated absorption peak at 1.52, where the absorption peak is caused by an H-bond interaction of H_(16)_-O_(13)_-C_(7)_-C_(3)_. Its vibration mode belongs to dihedral angle torsion. The simulated absorption peak of 1.27 THz is caused by an H-bond interaction of C_(55)_-H_(95)_, and its vibration mode is attributed to bond length stretch. The remaining peak points are attributed to intermolecular interaction. The experimental absorption peak of 4-HA at 0.83 THz corresponds to the simulated absorption peak at 0.84 THz, where the absorption peak is caused by the weak interaction between C_(1)_-C_(6)_-O_(78)_-C_(73)_. Its vibration mode belongs to dihedral angle torsion. The experimental absorption peak at 1.22 THz corresponds to the simulated absorption peak at 1.26 THz, where the absorption peak is caused by an H-bond interaction of H_(96)_-O_(60)_-C_(54)_, and its vibration mode is attributed to dihedral angle torsion. The simulated absorption peak at 1.49 THz is caused by the weak interaction between C_(3)_-C_(1)_-C_(6)_-O_(78)_, and its vibration mode is attributed to bond angle bend in plane. The experimental absorption peak at 1.83 THz corresponds to the simulated absorption peak at 1.76 THz, where the absorption peak is caused by intermolecular interaction.

### 2.3. Identifying Weak Interactions with IRI

**Figure 3:**
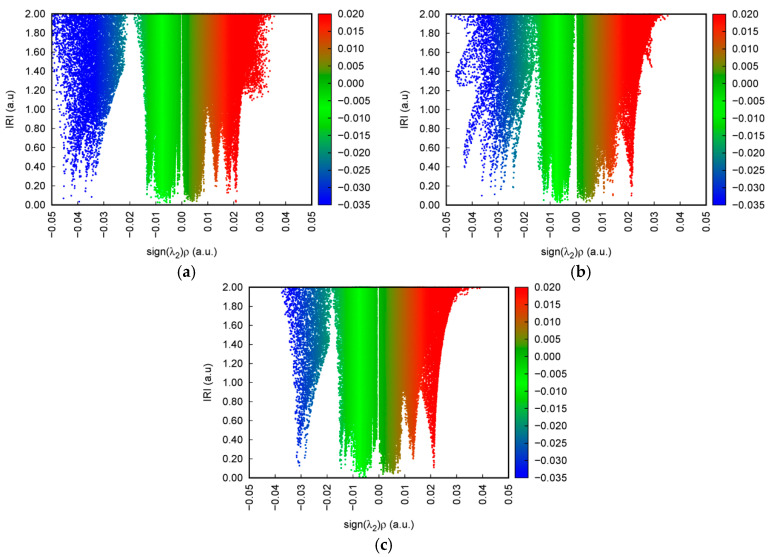
Scatter plots of the IRI versus the electron density multiplied by the sign of the second Hessian Eigen value (λ_2_) for (**a**) 2-HA cluster of seven molecules, (**b**) 3-HA cluster of seven molecules, and (**c**) 4-HA cluster of seven molecules. The surfaces are colored on a blue–green–red scale according to values of sign(λ_2_)*ρ* ranging from −0.035~0.02. Blue indicates stronger attractive interactions; red indicates stronger nonbonding overlap.

Figure 3 shows the IRI versus the electron density multiplied by the sign of the second Hessian Eigen value (λ_2_) for 2-, 3-, and 4-HA. The sign(λ_2_)*ρ* function can be mapped on IRI isosurfaces with different colors to vividly show nature of the interaction regions revealed by means of IRI. The sign(λ_2_) function denotes the sign of the second largest eigenvalue of the Hessian of *ρ*, which has certain ability to distinguish attractive and repulsive interactions. From the mapped colors of sign(λ_2_)*ρ*, one can easily identify the nature of interactions revealed by means of IRI isosurfaces. This coloring method will be employed for all figures given later. The density of the green dots in the scatter diagram is obviously greater than that of the blue dots and the red dots, which shows that the main weak interaction types of 2-, 3-, and 4-HA are vdW interactions, while H-bond and steric effect interactions account for a small proportion of the total weak molecular interactions.

**Figure 4:**
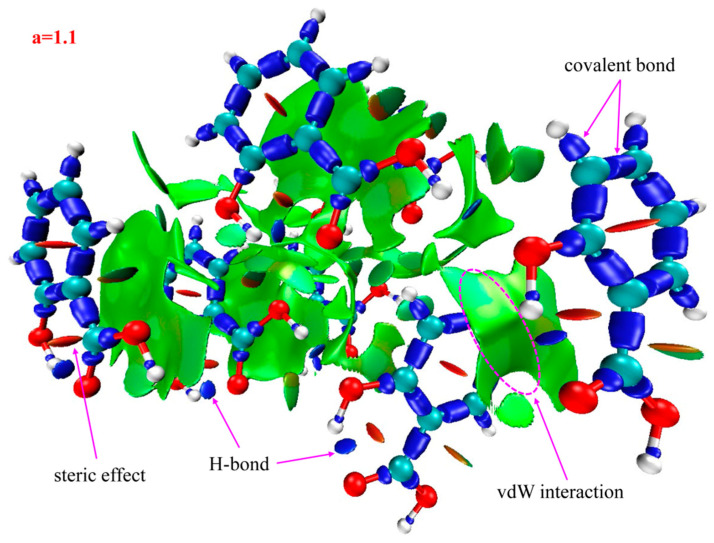
Isosurface map of a = 1.1 for 2-HA cluster of seven molecules. sign(λ_2_)*ρ* is mapped on the isosurfaces according to coloring method of Figure 3. Some featured regions in IRI map are labelled. Blue indicates stronger attractive interactions; red indicates stronger nonbonding overlap.

The spikes that this paper is interested in lie in the low-density, low gradient region, indicative of H-bond interactions in the system. The IRI isosurface for the 2-HA cluster of seven molecules is shown in Figure 4; multiple spikes are found in the region between −0.05 and −0.02 in Figure 3a. Combined with Table 2, the spikes can be divided into two points, i.e., −0.043 and −0.035, according to the corresponding values of sign(λ_2_)*ρ*. In addition, multiple spikes are found in the region between −0.02 and 0 which, belonging to vdW interactions, are located in −0.014, −0.009, −0.007, and −0.005; two spikes are found in the region between 0 and +0.05 which, belonging to steric effects, are located in +0.007 and +0.022.

**Figure 5:**
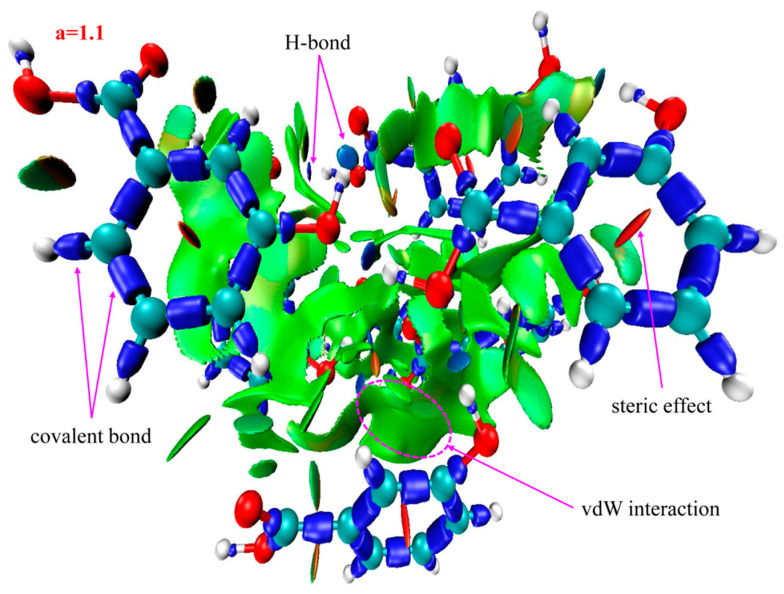
Isosurface map of a = 1.1 for 3-HA cluster of seven molecules. sign(λ_2_)*ρ* is mapped on the isosurfaces according to coloring method of Figure 3. Some featured regions in IRI map are labelled. Blue indicates stronger attractive interactions; red indicates stronger nonbonding overlap.

The IRI isosurface for the 3-HA cluster of seven molecules is shown in Figure 5. Belonging to H-bond interactions, multiple spikes are found in the region between −0.05 and −0.02 in Figure 3b. Associated with Table 2**,** these spikes can be divided into three points according to the corresponding values of sign(λ_2_)*ρ*, which are located in −0.043, −0.036, and −0.024. Moreover, two spikes are found in the region between −0.02 and 0, which, belonging to vdW interactions, are located in −0.012 and −0.006, and spikes belonging to steric effects are located in +0.004 and +0.021.

**Figure 6:**
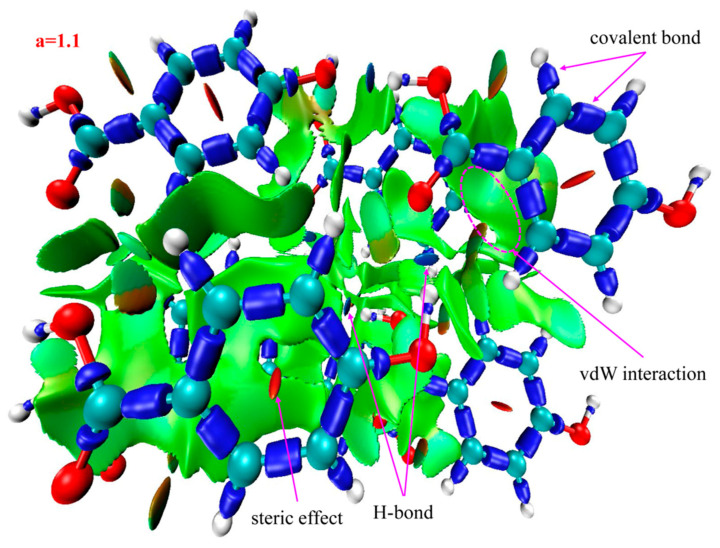
Isosurface map of a = 1.1 for 4-HA cluster of seven molecules. sign(λ_2_)*ρ* is mapped on the isosurfaces according to coloring method of Figure 3. Some featured regions in IRI map are labelled. Blue indicates stronger attractive interactions; red indicates stronger nonbonding overlap.

The IRI isosurface for the 4-HA cluster of seven molecules is shown in Figure 6. Belonging to H-bond interactions, one spike is found in the region between −0.05 and −0.02 in Figure 3c. The spike can be divided into one point according to the corresponding values of sign(λ_2_)*ρ*, located in −0.003 as seen in Table 2. In addition, two spikes are found in the region between −0.02 and 0, which belong to vdW interactions and are located in −0.014 and −0.006, and spikes belonging to steric effects are located in +0.006, +0.013, and +0.022.

Recalling the color scale bars mentioned in Figure 3, a conclusion could be made as follows: the highly green isosurfaces in Figure 4, Figure 5 and Figure 6 show that the intermolecular interactions of 2-, 3-, and 4-HA are dominated by the vdW interaction, respectively. At the same time, in the graphs, we also found some blue isosurfaces with a small area, which are regarded as weak H-bond interactions. In addition, we also found some red isosurfaces, as well as green and brown isosurfaces, all of which can be regarded as steric effects. In order to understand the results of the IRI method, Table 2 shows the relationship between weak interactions, isosurfaces, and peaks in the scatter plots.

Based on the above analysis, in general, the IRI method can display various types of intermolecular and intramolecular interactions at the same time and can be easily used alone to study weak intermolecular interactions. Because of the absorption of 2-, 3-, and 4-HA clusters in the THz band, it is the weak interaction between molecules that plays the leading role. From the results of IRI analysis, we observe that the isosurface is smooth and has no obvious defect, which effectively avoids unsightly jagged edges on the isosurface. The strength, location, and type of weak interaction can be displayed on the isosurface and scatter plot filled with BGR color. The results of IRI analysis for the three types of samples of 2-, 3-, and 4-HA are very similar to the scatter plots. The main weak interaction type of 2-, 3-, 4-HA is vdW interaction, and H-bond and steric interactions account for a small part of the total weak molecular interactions. It is confirmed that IRI analysis can provide valuable information for a deeper understanding of the structure and properties of molecules.

## 3. Materials and Methods

### 3.1. Experimental Apparatus

**Figure 7:**
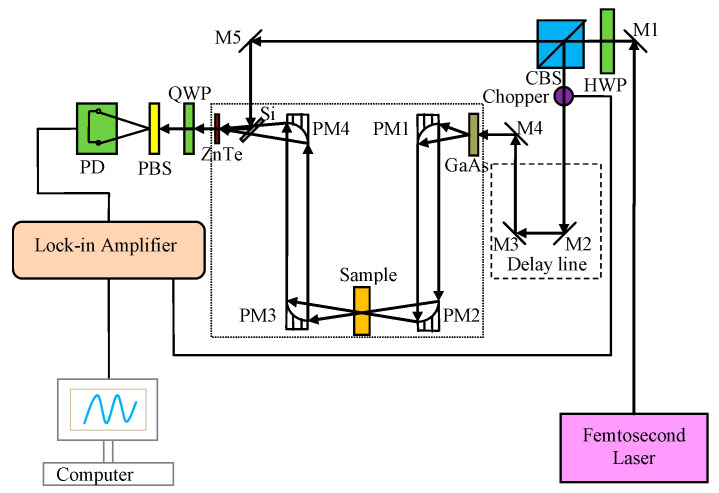
Schematic diagram of the Z-3 THz system [22].

The experimental setup consisted of a Z-3 THz system (repetition rate 82 MHz, pulse width: 100 fs, central wavelength: 780 nm, frequency resolution: ˂5 GHz(post fast Fourier transform (FFT)), signal-to-noise ratio: ˃70 dB; Zomega Terahertz Corp., New York, NY, USA). The schematic diagram of the system is shown in Figure 7. The apparatus is kept enclosed in a closed box that is purged with dry air (humidity: <2%, temperature: 295 K) to minimize the effect of water vapor present in the atmosphere [27]. The effective spectral range (Z-3 THz system) for obtaining stable spectral data was determined to be 0.6–2.0 THz.

### 3.2. Sample Preparation

**Figure 8:**
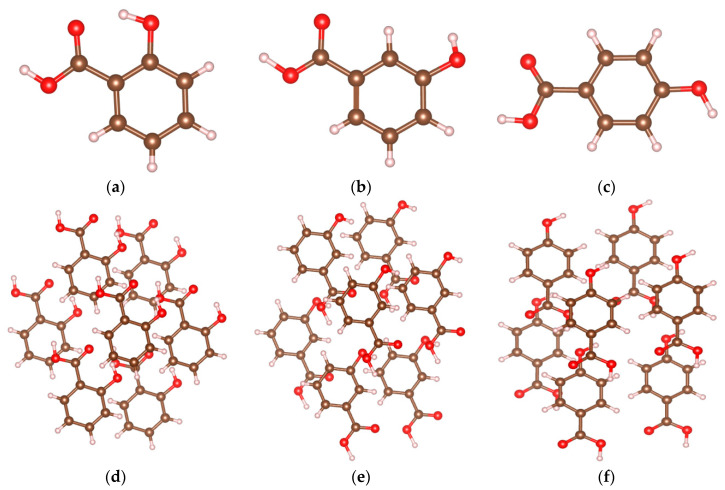
The molecular and cluster model of 2-, 3-, and 4-HA. Atomic elements in brown, peach, and red represent carbon, hydrogen, and oxygen atoms, respectively. These molecular structures are displayed using VESTA software. (**a**) Molecular of 2-HA; (**b**) molecular of 3-HA; (**c**) molecular of 4-HA; (**d**) cluster model of 2-HA; (**e**) cluster model of 3-HA; (**f**) cluster model of 4-HA.

The samples (2-, 3-, and 4-HA) in the paper were purchased from Shanghai Aladdin Biochemical Technology Co., Ltd., Shanghai, China. The molecular formulae of the hydroxybenzoic acids are shown in Figure 8. The purity of 2-HA was 99.5%, that of 3-HA was 98.0%, and that of 4-HA was 99.0%. Three highly pure compounds were used for the experiment without further purification. The samples were not ground as they were in powdered form. The samples were screened through a 180-mesh sieve, and then were compressed with approximately 10 MPa pressure. Subsequently, the samples were dried at a temperature of 50 °C for approximately 2 h. After completing these steps, samples with diameters of approximately 13 mm and thicknesses of approximately 1 mm were obtained. Among the samples, crack-less samples with smooth surfaces were selected as the test samples to record the spectra [28].

### 3.3. Computational Details

The 2-, 3-, and 4-HA samples were analyzed using the Z-3 THz system. The absorption spectra of the three samples were recorded as the experimental comparison objects for the identification of the characteristic peaks. Specifically, the time domain signals measured using the Z-3 THz system were transformed using the FFT algorithm to obtain the frequency domain spectrum; we then calculated the absorbance with the following formula [29]:

(1) $$\mathrm{Absorbance}=-\mathrm{lg}\left[\frac{E^{2}{}_{trans}(\omega )}{E^{2}{}_{0}(\omega )}\right],$$

where *E*_0_(*ω*) is the THz amplitude of the reference, *E_trsns_*(*ω*) is the THz amplitude of the sample, and ω is the circular frequency.

The Thomas–Fermi model, based on DFT theory, divides the entire system space into sufficiently small cubes and obtains the corresponding energy and density via solving the Schrodinger equation of the particles in the infinite potential well of any cube (without considering the interaction between electrons) [30]:

(2) $$E_{TF}=c_{F}\int \rho^{5/3}(r)dr-z\int \frac{\rho (r)}{r}dr+\frac{1}{2}\iint \frac{\rho_{1}(r)\rho_{2}(r)}{\left|r_{1}-r_{2}\right|}dr_{1}dr_{2},$$

In Formula (2), only the interactions between nucleus and electron and electron and electron are considered. *c_F_* = 2.871, *ρ*(*r*) is the electron density, and *E_TF_* is the total kinetic energy. If *ρ*_0_(*r*) is the electron density distribution of the definite system, then *E*(*ρ*_0_) is the lowest energy, that is, the ground state energy of the system; then, for a definite electron system, given the crystal field potential energy *ν*(*r*), there exists a functional *Eν*[(*ρ*(*r*))] of *ρ*(*r*) that satisfies the following principles:

(3) $$E_{\nu }[\rho (r)]\geq E[\rho_{0}(r)]=E_{0},$$

The electron density can usually be expressed in the form of an N-problem orbital, and the expressions of energy and density can be transformed into the following form:

(4) $$E_{\nu }=\int \left[\rho (r)\nu (r\right)]dr+\frac{1}{2}\int \frac{1}{\left|r-r^{\prime }\right|}\rho (r)\rho (r^{\prime })drdr^{\prime }+T(\rho )+E_{xc}(\rho ),$$

In Formula (4), the first term is the interaction energy of the nucleus and the electron, the second term is the electrostatic repulsion, the third term is the kinetic energy functional, and the fourth term is the system exchange-correlation functional. The sum of the first term and the second term is the classical Coulomb action. The focus of this project is on the third and fourth items.

In order to calculate and optimize the cluster structures of hydroxybenzoic acid isomers using DFT, their optimal cell models need to be constructed. The cell configurations of 2-HA, 3-HA, and 4-HA were obtained from the Cambridge Crystallographic Data Centre (CCDC), Cambridge, UK [31], and in order to match the three isomeric crystal models with the actual substances, the model construction principles were based on the hydroxybenzoic acid molecular cluster [32]. Based on the open-source crystal models of the three isomers, the GaussView16 software and visual molecular dynamics software were used to extend the periodically arranged complex crystals, and then keyed to the cluster structure of a single molecule surrounded by six neighboring molecules. Since the structure of the complex cell is uniquely determined, the final 7-molecule cluster structure generated is also the only determined optimal structure. In order to ensure that the results of the theoretical calculations reach a more desirable accuracy based on the allowed computational cost, the relatively novel contemporary B3LYP hybridization generalization and 6-311G++(d, p) basis group levels are used, and D3 dispersion correction is added to better describe the dispersion effect [33]. The temperature and air pressure settings for the theoretical calculations are 298.15 K and 1 atm, respectively.

*IRI* has the same effect in showing weak interactions as well as the reduced density gradient (RDG). A key advantage of *IRI* is that under the same isosurface value in the chemical bond zone, it can show the interactions of different strengths at the same time. Clearly, it is possible to graphically investigate all types of interactions in the system at a glance. This advantage is very meaningful when analyzing weak interactions between molecules. The *IRI* function is simply defined as [34]:

(5) $$IRI(r)=\frac{\left|\nabla \rho (r)\right|}{{\left[\rho (r)\right]}^{a}}$$

where *ρ*(*r*) is the electron density and a is an adjustable parameter (*a* = 1.1 is adopted for standard definition of *IRI*). *IRI* is essentially the gradient norm of electron density weighted by scaled electron density. The graphics of hydrogen bonding interactions were obtained using VMD1.9.3 software [35].

**Figure 9:**
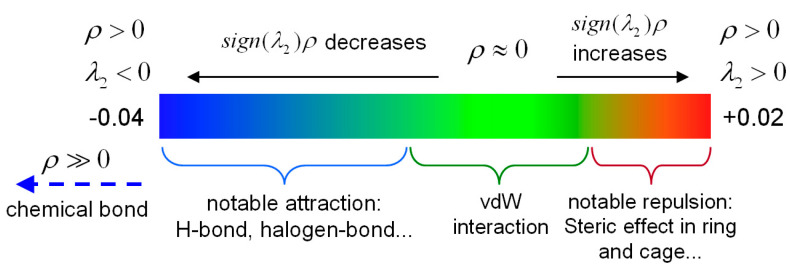
Description of the weak interactions based on the color scale.

In order to analyze the properties of intermolecular interactions in 2-, 3-, and 4-HA crystals, *IRI* analysis was carried out with Multiwfn software [36]. Visualization of noncovalent interaction regions was performed using the Multiwfn3.8 program. Figure 9 shows the isosurface map used to determine the type of intermolecular interaction in the *IRI* visual analysis. The blue area indicates hydrogen bond (H-bond) interaction, while the green area is van der Waals (vdW) interaction, and the red area means the steric effect interaction. If the color of the isosurface is obviously reddish, it means that there is a positioning hindrance. If it is bright red, it means that the steric hindrance is very strong. If the color of the isosurface is obviously bluish, it indicates that there is a significant attraction, such as hydrogen bonds and halogen bonds of general strength. If the isosurface is completely blue, it means that there is either a relatively strong weak interaction (the electron density in the action area can reach ≥0.04 a.u.), or a covalent bonding interaction where the electron density in the bonding region is usually significantly larger than 0.04 a.u. [37].

Furthermore, the sign(λ2)ρ function was defined in the IRI method, which was calculated using the actual electron density. Hence, when the sign(λ2)ρ function was mapped on the isosurfaces with a blue–green–red (BGR) color scale, a clear view of the interaction region and interaction type were obtained.

## 4. Conclusions

The THz vibrational absorption spectra of the three positional isomers of 2-, 3-, and 4-HA were reported in the range of 0.6–2.0 THz at room temperature through THz-TDS. At least three significant absorption peaks were obtained at different positions; the peaks are the “signature fingerprints” of organic molecular isomers. This shows that THz spectroscopy technology can be used as a new method to identify subtle differences in material structure. The vibrational spectra of 2-, 3-, and 4-HA seven-molecular clusters were simulated with DFT-D3 calculations, and the results showed that the simulated spectra were basically consistent with the experimental spectra. In order to study the absorption mechanism of organic molecules using THz waves, PED analysis was performed on the simulation data, and the vibration modes corresponding to the absorption peaks of 2-, 3-, and 4-HA were determined. IRI analysis qualitatively reveals the type of weak interaction between molecules, and it showed that the weak interaction type of 2-, 3-, and 4-HA is dominated by vdW interactions. It may have potential scientific value for understanding the structure of matter and predicting the physical and chemical properties of materials because H-bond and steric effect interactions are both small. More importantly, this will help promote the development of biopharmaceuticals and chemical synthesis.

## Figures and Tables

**Table 1:** Vibrational modes description assigned to each absorption peak of the hydroxybenzoic acids.

Samples	Experimentally Obtained/THz	Calculated/THz	Vibrational Modes
2-HA	1.12	1.03	O: O_(1)_H_(16)_ and C_(25)_C_(21)_H_(16)_
	1.41	1.33	B: H_(48)_C_(53)_C_(57)_; T: C_(10)_C_(103)_O_(98)_H_(87)_
	–	1.78	Intermolecular interaction
	1.97	–	Intermolecular interaction
3-HA	0.81	0.85	Intermolecular interaction
	1.12	–	Intermolecular interaction
	–	1.27	S: C_(55)_H_(95)_
	1.48	1.52	T: H_(16)_O_(13)_C_(7)_C_(3)_
	1.81	1.85	Intermolecular interaction
4-HA	0.83	0.84	T: C_(1)_C_(6)_O_(78)_C_(73)_
	1.22	1.26	B: H_(96)_O_(60)_C_(54)_
	–	1.49	T: C_(3)_C_(1)_C_(6)_O_(78)_
	1.83	1.76	Intermolecular interaction

Notes: Analysis software output parameter with bond angle bend in plane (B), dihedral angle torsion (T), bond length stretch (S), and out-of-plane angle bending (O) vibrational modes are abbreviated.

**Table 2:** Intermolecular and intramolecular weak interaction description of 2-, 3-, and 4-HA clusters.

Samples	Spikes/a.u.	Isosurfaces	Weak Interaction Categories
2-HA	sign(λ_2_)ρ = −0.043 sign(λ_2_)ρ = −0.035	blue	H-bonds
	sign(λ_2_)ρ = −0.014 sign(λ_2_)ρ = −0.009 sign(λ_2_)ρ = −0.007 sign(λ_2_)ρ = −0.005	green	vdW interactions
	sign(λ_2_)ρ = +0.007 sign(λ_2_)ρ = +0.022	brown	steric effects
		red	
3-HA	sign(λ_2_)ρ = −0.044 sign(λ_2_)ρ = −0.036 sign(λ_2_)ρ = −0.024	blue	H-bonds
	sign(λ_2_)ρ = −0.012 sign(λ_2_)ρ = −0.006	green	vdW interactions
	sign(λ_2_)ρ = +0.004 sign(λ_2_)ρ = +0.021	brown red	steric effects
4-HA	sign(λ_2_)ρ = −0.03	blue	H-bonds
	sign(λ_2_)ρ = −0.014 sign(λ_2_)ρ = −0.006	green	vdW interaction
	sign(λ_2_)ρ = +0.006 sign(λ_2_)ρ = +0.013 sign(λ_2_)ρ = +0.022	brown	steric effects
		red	

Notes: Spikes belong to the scatter plots (Figure 3). Isosurfaces belong to the isosurface map (Figure 4, Figure 5 and Figure 6).

## Data Availability

Any data or material that support the findings of this study can be made available by the corresponding author upon request.
